# Spider foraging strategy affects trophic cascades under natural and drought conditions

**DOI:** 10.1038/srep12396

**Published:** 2015-07-23

**Authors:** Shengjie Liu, Jin Chen, Wenjin Gan, Douglas Schaefer, Jianmin Gan, Xiaodong Yang

**Affiliations:** 1 Key Laboratory of Tropical Forest Ecology, Xishuangbanna Tropical Botanical Garden, Chinese Academy of Sciences, Mengla, Yunnan 666303, China; 2 Key Laboratory of Vegetation Restoration and Management of Degraded Ecosystems, South China Botanical Garden, Chinese Academy of Sciences, Guangzhou 510650, China

## Abstract

Spiders can cause trophic cascades affecting litter decomposition rates. However, it remains unclear how spiders with different foraging strategies influence faunal communities, or present cascading effects on decomposition. Furthermore, increased dry periods predicted in future climates will likely have important consequences for trophic interactions in detritus-based food webs. We investigated independent and interactive effects of spider predation and drought on litter decomposition in a tropical forest floor. We manipulated densities of dominant spiders with actively hunting or sit-and-wait foraging strategies in microcosms which mimicked the tropical-forest floor. We found a positive trophic cascade on litter decomposition was triggered by actively hunting spiders under ambient rainfall, but sit-and-wait spiders did not cause this. The drought treatment reversed the effect of actively hunting spiders on litter decomposition. Under drought conditions, we observed negative trophic cascade effects on litter decomposition in all three spider treatments. Thus, reduced rainfall can alter predator-induced indirect effects on lower trophic levels and ecosystem processes, and is an example of how such changes may alter trophic cascades in detritus-based webs of tropical forests.

Predation has important roles in the structure and function of ecological systems^1^. Ecosystem services provided by predators include directly controlling the abundances of herbivores that are potential agricultural pests^2^,^3^,^4^, and indirect effects on ecosystem functions such as primary productivity and elemental cycling through trophic cascades^5^,^6^,^7^,^8^. Trophic cascades, the indirect effects of predators on non-adjacent trophic levels, have long been recognized in the ecological literature^3^,^8^. As previously reviewed, strengths of predator trophic cascades depends on herbivore species diversity, predator type (vertebrate stronger than invertebrate), ecosystem type (terrestrial stronger than aquatic systems), predator metabolic factors (predators associated with the strongest cascades were endothermic vertebrates) and primary productivity (high system productivity stronger than low system productivity)^9^,^10^,^11^.

In terrestrial ecosystems, predator impacts on ecosystems can strongly depend on their functional traits, including morphology, microhabitat use and foraging behaviors^12^,^13^,^14^,^15^. Previous studies reveal that actively hunting spiders directly decrease the number of herbivore prey by capturing and consuming them, and indirectly reduce plant species diversity and enhance aboveground net primary production^8^. In contrast, sit-and-wait spiders largely cause behavioral responses in herbivore prey, and indirectly have positive cascading effects on species composition of plant communities^8^,^11^. These different effects are related to a single functional trait, predator foraging strategy, irrespective of taxonomic identity^1^. Roles of predator foraging strategies have been examined most often in food webs based on living plant matter^10^. Effects of predator foraging strategies in detritus-based food webs have been less explored^10^.

Theory and accumulating empirical evidence both suggest that predators in detrital food webs may initiate trophic cascades affecting decomposition rates, particularly through the fungal channel of decomposer food webs^16^,^17^,^18^,^19^. One such pathway may involve spiders and soil macrofauna and mesofauna^16^. Spiders represent a large fraction of arthropod predators in forests, and spiders have high diversity in tropical forest floors^20^,^21^. Spiders in tropical forest floors have two common distinct foraging strategies: one is wandering (species that do not build webs to capture prey) which is an actively hunting predator; the other is web-building spider, which is one kind of sit-and-wait predator with continuous presence at fixed locations^8^. The potential prey of epigeous (occupying the forest floor) spiders consists mainly of Collembola, Diptera, Coleoptera and Hymenoptera (Supplementary Fig. S1)^21^. Field experiments have shown that spiders affect decomposition rates by limiting microbi-detritivore densities in a deciduous forest floor^17^,^22^. However, it remains unclear how spiders’ foraging strategies influence soil fauna communities and, consequently, have cascading effects on litter decomposition rates in tropical systems.

Precipitation patterns have shifted over the 20^th^ century and climate-change predictions suggest that droughts could become more frequent and intense in Yunnan, southwestern China^23^,^24^. Xishuangbanna (southern Yunnan) is located in one of 25 biodiversity hotspots in the world^25^. During the decade from 2002 to 2012, Xishuangbanna experienced severe droughts in 2003, 2004, 2009, 2010 and 2011 (Supplementary Fig. S2A). The climate of Xishuangbanna is representative of the Asian monsoon with only 10% to 15% of annual precipitation during the dry season from November to April. (Supplementary Fig. S2B). Changes in precipitation amounts can directly alter soil moisture which strongly influences soil fauna reproduction and development rates^26^. Furthermore, drought can directly modify soil fauna community composition and abundance by altering soil microclimate, and indirectly by altering resource availability and composition of the soil food web^27^,^28^,^29^. Soil fauna are also extremely responsive to changes in soil moisture across diverse ecosystems^27^,^28^,^29^,^30^. Therefore, increased dry periods should have important consequences for trophic interactions in detritus-based food webs and forest floor ecosystems.

In this study, we manipulated densities of spiders with two different foraging strategies, actively hunting (AH) and sit-and-wait (SW), in microcosms mimicking the tropical rainforest floor ecosystem, and asked what are: (1) the influences of two different spider types on soil fauna abundance in tropical forest floor; (2) the potential trophic cascade effects of these spider types on rates of litter decomposition; (3) the consequences of moisture reduction on direct and cascading effects of two different spider foraging strategies in a detrital food web.

## Results

### Initial density of soil fauna communities and soil moisture

Initial soil faunal densities among the four treatments used for natural and reduced rainfall manipulations did not differ (all *P* > 0.05) (Supplementary Table S1). The rainout shelters limited water input to microcosms and reduced soil moisture (*P* < 0.001) (Supplementary Fig. S3).

### Comparison of soil fauna abundance among different spider treatments under ambient and drought conditions

**Figure 1:**
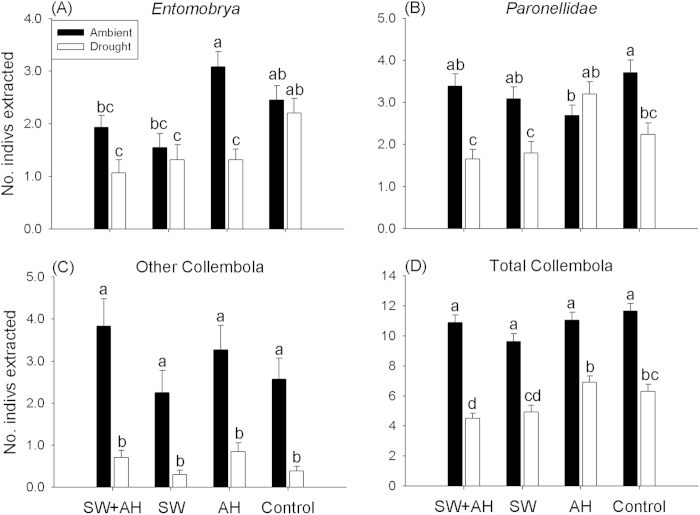
Effects of different spider treatments on the abundance of main groups of Collembola under ambient and drought conditions. (**A**) *Entomobrya*, (**B**) *Paronellidae*, (**C**) Other Collembola, D) Total Collembola. Data are expressed as mean ± s.e.m (n = 10). Means with different letters are significantly different (*P* < 0.05). Note that *y*-axes have different scales.

Repeated-measure GLMMs showed that interactions between spider and rainfall treatments had significant effects on the abundance of various soil fauna (all *P* < 0.05) (Supplementary Table S2), thus we separately compared these groups under drought and ambient conditions. The taxonomic groups of Collembola responded differently to spider treatments. Under ambient conditions, *Entomobrya* abundance was lower in SW + AH and SW compared to AH (*P* = 0.01, *P* = 0.004, respectively), and there were no differences among the other spider treatments and control (all spiders excluded, as below) (all *P* > 0.05) (Fig. 1A). The AH spider treatment significantly reduced *Paronellidae* abundance compared to the control (*P* = 0.04) (Fig. 1B). We observed no differences in abundance of Other Collembola or Total Collembola across the four treatments under ambient conditions (all *P* > 0.50) (Fig. 1C,D). For the Acari group, which were not potential prey of these spiders, we unexpectedly found that AH had the highest Oribatid abundance (Table 1).

**Figure 2:**
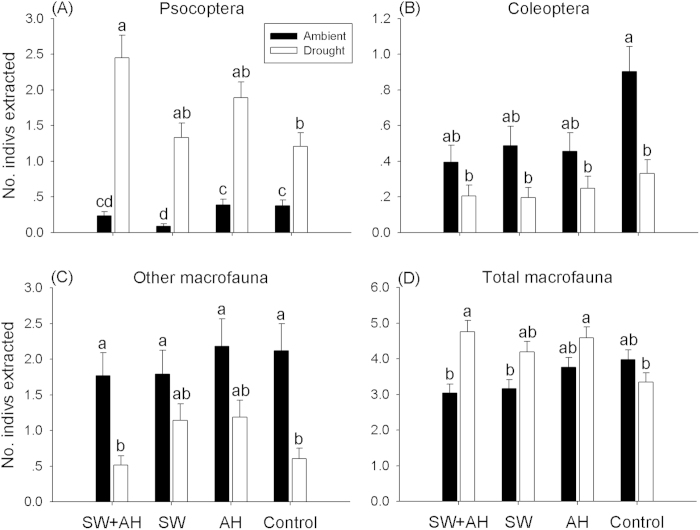
Effects of different spider treatments on the abundance of main groups of macrofauna under ambient and drought conditions. A) Psocoptera, B) Coleoptera, C) Other Macrofauna, and D) Total Macrofauna. Data are expressed as mean ± s.e.m (n = 10). Means with different letters are significantly different (*P* < 0.05). Note that *y*-axes have different scales.

For soil macrofauna, taxonomic groups also responded differently to spider treatments under ambient conditions. Higher abundance of Psocoptera was found in control and AH than in SW (*P* = 0.017, *P* = 0.02, respectively) (Fig. 2A). There were no differences in Coleoptera, Other macrofauna or Total macrofauna abundance across the spider treatments (all *P* > 0.05) (Fig. 2 B–D).

Under drought conditions, the control treatment had higher *Entomobrya* abundance than SW + AH, SW or AH treatments (*P* = 0.01, *P* = 0.03, *P* = 0.01, respectively) (Fig. 1A). For *Paronellidae* abundance, AH was significant higher than SW and SW + AH (*P* = 0.04, *P* = 0.03, respectively), while the control treatment did not differ from any spider treatment (all *P* > 0.05) (Fig. 1B). We observed no differences in abundance of Other Collembola across the four treatments (all *P* > 0.30) (Fig. 1C). For Total Collembola abundance, SW + AH was significantly lower than AH and control treatment (*P* = 0.02, *P* = 0.04, respectively) (Fig. 1D).

For macrofauna, repeated-measured GLMM revealed that Psocoptera abundance in SW + AH was higher than in the control (*P* = 0.02), while there were no differences between SW or AH and control (*P* = 0.84, *P* = 0.53, respectively) (Fig. 2A). We observed no differences in abundance of Coleoptera or Other Macrofauna among treatments (Fig. 2B,C). Abundance of Total Macrofauna was higher in AH and SW + AH than in control treatment, mostly due to more Psocoptera (*P* = 0.01, *P* = 0.047, respectively) (Fig. 2D).

### Effects of drought on soil fauna abundances across the four spider treatments

Most soil fauna were more abundant in ambient than drought microcosms, but Psocoptera showed the opposite pattern (Fig. 2A). Among Collembola, drought significantly decreased abundance of Other Collembola and Total Collembola compared with ambient treatment across all spider treatments (Fig. 1C,D). For *Entomobrya*, drought only had significant effects in AH (*P* < 0.001) (Fig. 1A), and drought had significant effects on *Paronellidae* in three spider treatments other than AH (Fig. 1B). For macrofauna, ambient-moisture microcosms had more Coleoptera than drought microcosms in control treatment (*P* = 0.002) (Fig. 2B), and ambient microcosms had higher Other Macrofauna abundance than drought microcosms in SW + AH and control treatments (*P* = 0.01, *P* = 0.006, respectively) (Fig. 2C) (see more detail in Fig. 2). Moreover, drought had no significant effects on Oribatids or Other Acari, not potential prey of these spiders, in the SW treatment (Table 1).

### Trophic cascade index

**Figure 3:**
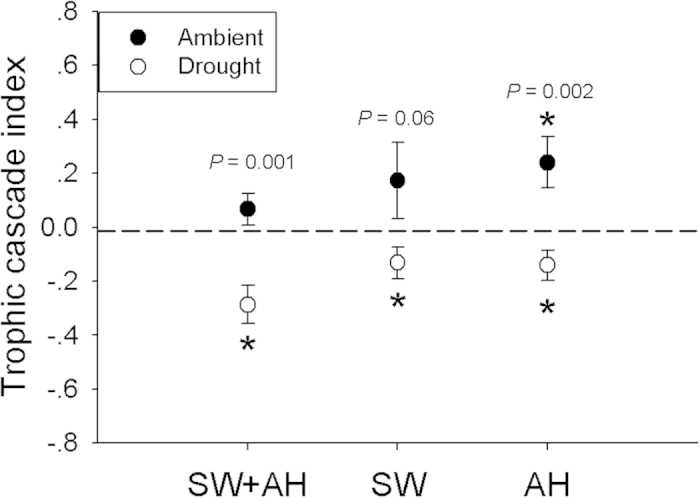
Effects of spider-induced trophic cascade on leaf litter decomposition rate expressed as trophic cascade index under ambient and drought condition. A value of trophic cascade index above the dotted line (zero) indicates positive effects of treatments on litter decomposition, near the dotted line (zero) indicates the absence of trophic cascade, and below the dotted line (zero) indicates negative effects of treatments on litter decomposition. Asterisks indicate significance differences between index and dotted line (zero) (*P* < 0.05, Bonferroni-corrected). Data are expressed as mean ± s.e.m (n = 10). The P value indicate the differences of trophic cascade index between ambient and drought conditions.

A positive trophic cascade index (above the dotted line) indicates positive treatment effects on litter decomposition, near the dotted zero line indicates the absence of trophic cascades, and below zero indicates negative treatment effects on litter decomposition rates (Fig. 3). Under ambient moisture conditions, the trophic indices of SW + AH and SW were close to zero (t = 1.13, *P* = 0.29; t = 1.23, *P* = 0.25, respectively), indicating no cascading effect on decomposition rates (Fig. 3). In contrast, AH significantly accelerated decomposition rates (t = 2.53, *P* = 0.03). Under drought conditions, all of three spider treatments reduced decomposition rates (SW + AH: t = −4.05, *P* = 0.003; SW: t = −2.22, *P* = 0.05; AH: t = −2.25, *P* = 0.03) (Fig. 3). Comparing trophic cascade indices in ambient and drought among the spider treatments showed that ambient microcosms had higher cascading effects than drought microcosms in SW + AH and AH spider treatments (SW + AH: t = −3.94, *P* = 0.001; AH: t = -3.68, *P* = 0.002). There were no differences between ambient and drought microcosms in the SW treatment (t = −2.00, *P* = 0.06) (Fig. 3).

## Discussion

We found a positive trophic cascade on litter decomposition rates triggered by actively hunting (AH) spiders under ambient moisture, which could be explained by their indirect positive effects on Oribatid abundance. However, sit-and-wait (SW) spiders had no effect on the soil fauna except Psocoptera, thus SW spiders had no cascading effects on litter decomposition under ambient moisture. In contrast, drought reversed the cascading effects of spiders on litter decomposition rates. Under drought conditions, we observed negative trophic cascade effects on litter decomposition in all three spider treatments, and decreased *Entomobrya* densities.

In agreement with Lawrence and Wise^18^, we found that actively hunting spiders could indirectly increase decomposition rates (Supplementary Fig. S4). While AH spider treatment decreased the density of *Paronellidae* under ambient-moisture conditions, previous studies indicated that various Collembola can have markedly different effects on litter decomposition, with functional redundancy among common species in soil ecosystems^31^,^32^,^33^. *Paronellidae* may have minor roles in litter decomposition in our system. In contrast, AH spider treatment had higher Oribatid density compared with control treatment, and they are key decomposers of soil organic matter^34^,^35^. It is possible that increased Oribatidae enhanced decomposition rates under ambient conditions, and spiders may have suppressed populations of major predators on Oribatids (for example, Mesostigmatid mites^36^). Indirect effects mediated through other microbi-detritivores might also have contributed to increased numbers of Oribatid mites^18^.

In contrast, SW + AH and SW spider treatments did not show cascading effects on litter decomposition under ambient moisture. The density of Total Macrofauna and Collembola in SW + AH and SW were not different from those in control treatment, suggesting that macrofauna and Collembola have similar effects on litter decomposition rates in these three treatments under ambient conditions (Supplementary Fig. S4). Unexpectedly, we found that the SW spider treatment had lower densities of Psocoptera. Because Psocoptera populations were always small, they may have little effect on litter decomposition rates. We suggest that sit-and-wait spiders at natural densities had no significant density effect (lethal effect) on soil fauna, thus did not cause cascading effects on litter decomposition rates^37^. A possible reason is that sit-and-wait predators cause largely evasive behavioral responses in their prey because prey species respond more strongly to persistent, point-source cues of predator presence^8^.

We detected negative trophic-cascade effects on decomposition rates with spiders under drought conditions. Compared to control, SW + AH, SW and AH spider treatments significantly decreased densities of *Entomobrya*, and thus slowed litter decomposition. *Entomobrya* are relative large, surface-dwelling Collembola acting as primary decomposers by feeding directly on litter and its associated fungi and bacteria, thus playing essential roles in litter decomposition^16^,^38^. Meanwhile, SW + AH had lower densities of Total Collembola compared with control under drought conditions, and Collembola in general are major drivers of litter decomposition in tropical forests^39^. Drought can indirectly increase the rate of Collembola movement by affecting availability of their fungal food. More fungi in wetter areas may contribute to increased Collembolan emigration from dry areas^40^. Sit-and-wait spiders are more likely to encounter and consume mobile *Entomobrya* associated with environment change such as drought. Our findings are consistent with previous studies: that presence of spider could decrease rates of decomposition, accompanied by decreased in Collembola (mainly *Entomobrya*) densities in field experiments^16^,^17^.

The trophic cascade index in SW + AH and AH spider treatments under drought was significantly lower than that index under ambient moisture, indicating that drought significantly reduced trophic cascade indices in SW + AH and AH spider treatments. Drought significantly decreased abundances of Other Collembola and Total Collembola compared with ambient conditions in SW + AH and AH spider treatments. Decreased moisture can lead to lower Collembola densities by reducing the growth of fungi, a main food source of Collembola^41^. However, for Total Macrofauna, SW + AH effects under drought were higher than that ambient-moisture, due largely to higher numbers of Psocoptera. Psocoptera abundance may increase during droughts^42^. There have been few studies on the effects of Psocoptera on decomposition rate, but one found that Psocoptera occurred only in late stages of decomposition^42^, thus their effects may be minor. In contrast, drought did not change the trophic cascade index in the SW spider treatment as comparing with ambient moisture. One possible reason is that although sit-and-wait spider decreased density of Total Collembola, there were no differences in Oribatidae and Other Acari between drought and ambient moisture in the SW spider treatment. As mentioned above, Oribatids were a major faunal element enhancing decomposition^34^. Another possible explanation for the different responses of the two different spiders is that AH spider probably has stronger capacity of enduring desiccation^21^, which indicate that AH spiders cause stronger predation pressure on those soil fauna than SW spiders. This experiment provides evidence that reduced rainfall decreases abundance of some soil fauna in a detrital food web, which should enhance the strength of cascading effects by predators in tropical forest floors.

In conclusion, our study revealed that different spider-foraging strategies had different trophic cascade effects under ambient rainfall, but spiders with both foraging strategies slowed litter decomposition under drought conditions. Furthermore, changes in soil-moisture content can affect interactions between soil fauna and spiders in the detrital food web by altering population densities and activity of soil fauna in tropical forest floors. As a consequence, understanding how increased dry periods affect the role of biota in ecosystem processes such as litter decomposition and nutrient return to forests is important for predicting tropical forest-floor responses to global climate change.

## Methods

### Studied spiders

*Macrothele yunnanica* (Hexathelidae) and *Pardosa laura* (Lycosidae) are dominant spiders with regard to biomass and density in this study site, and exhibit two different foraging strategies. A sit-and-wait predator, *M. yunnanica* can live for about two years with a continuous presence at a fixed habitat location. It thus may provide a persistent point-source cue of high risk to prey^1^,^43^,^44^. These spiders typically build silk-lined tubular burrow retreats with open funnel entrances from which irregular trip-lines radiate over the litter layer. The actively hunting predator, *P. laura* can live for about eight months, and roams widely on the forest floor in search of prey^45^. An earlier experiment (Shengjie Liu, unpublished data) indicated that of these two dominant species, only AH exerted significant predation pressure on Collembola (Supplementary Fig. S5).

### Experimental soil and litter

This experiment was conducted in replicated microcosms. Soils were collected from the mineral layer (0 to 20 cm) in January 2012 from a tropical secondary forest located in Xishuangbanna Tropical Botanical Garden (XTBG), Chinese Academy of Sciences (101°11’ E, 21°56’ N, see detail in Yang and Chen^46^). After sampling, soils were sieved through 5 mm mesh to remove rocks and roots and then thoroughly homogenized. The soil was a Oxisol, with a soil organic matter content of 39.03 g kg^−1^ and total N of 2.29 g kg^−1^. Litter and humus were collected from same site as soil samples in March 2012. After those collections, large spiders were first removed by hand, and then litter was carefully sifted through a 3-mm mesh screen, removing all spiders encountered, as well as twigs and rocks. All non-spider fauna were replaced and then the litter was fully mixed. This litter and humus hosted the ambient faunal community with a natural level of soil fauna density and biomass. The 20 to 25 m canopy of the tropical secondary forest is composed mainly of species of *Pometia tomentosa*, *Litsea glutinouse*, *Castanopsis indica*, *Phoebe lanceolata,* and *Schefflera venulosa*. More detailed descriptions of geomorphology, vegetation and soil in our study site can be found in Zhang and Cao^47^.

### Microcosm preparation

Microcosms were prepared in plastic pots (60 cm diameter and 20 cm height) and filled with 25 kg of fresh-weight-equivalent soil and 250 g fresh litter and humus. The mean thickness of the soil and litter layers were about 12 cm and 1.5 cm, respectively. Drainage holes in the bottom of the pots were covered with 1 mm mesh before soil was added. The above operations were disruptive, so all microcosms were placed in a secondary forest for one month, allowing time for recovery. During that time, microcosms were periodically moistened, and covered with 1-mm mesh fiberglass screen to limit immigration of spiders. One month later, we added healthy adult female spider to create microcosm treatments (see below). Spiders were collected locally from this forest. Microcosms were then again covered with 1-mm mesh fiberglass screen to limit migration of spiders and soil fauna.

We used litterbags to evaluate rates of litter disappearance^48^. We selected single-species *Pometia tomentosa* (leaf litter C: N = 42.5), a dominant plant species in this forest, as an indicator of the potential of spiders to influence litter decomposition. Its leaf litter was collected locally from our sampled tropical forest floor and left to air dry for two weeks before use. Three grams of air-dried *P. tomentosa* leaf litter was placed in 10 cm × 10 cm litter bags with 2 mm mesh, which were then placed on the soil surface inside the prepared microcosms. We selected 2-mm nylon mesh bags according to the body size (width) of litter fauna. Most macro, meso, and microfauna can enter the 2-mm bags^48^. Six litter bags were set below the litter and attached to the soil surface in each microcosm. To avoid impacting spider activity, we installed those litter bags before adding spiders. All microcosms were kept in the same tropical secondary forest floor throughout the experiment. To each microcosm, we added leaf litter every month to match the natural monthly litter fall (Supplementary Table S3). That litter was collected from the same forest by suspended litter traps, and fauna were removed from it by a freeze-thaw cycle.

### Experimental design

Microcosms were placed on the forest floor in a randomized block design with ten treatment blocks. Each block, consisting of one replicate per treatment, had at least 5 m spacing. Within blocks, we randomly divided the microcosms into two rainfall treatments: ambient or drought. Four microcosms in each block were uncovered, unmanipulated and received natural rainfall (ambient treatment). Rain shelters, 1.5 m above the microcosms, were constructed over the four drought microcosms using PVC pipes and transparent polyethylene sheeting. The roofs over four microcosms excluded rainfall to simulate severe drought conditions (drought treatment), but allowed natural ventilation. Similar extreme changes in rainfall are predicted to occur with global warming^24^,^25^. Then we randomly assigned microcosm in each block to one of four spider treatments: Control: where no spiders were added; SW spiders: where 4 adult female *M*. *yunnanica* spiders were maintained in the microcosms; AH spiders: where 4 adult female *P*. *laura* spiders were kept in the microcosms; SW + AH spider: where 2 adult female *M*. *yunnanica* and 2 adult female *P*. *laura* spiders were kept in the microcosms. These densities were within the range (*M*. *yunnanica*: 2–12 individual/m^2^, average value 6 individual/m^2^; *P*. *laura*: 1-9 individual/m^2^, average value 6 individual/m^2^) observed in the field. During this one-year experiment, we checked spider abundances two or three times per month and replenished them as required (Supplementary Table S4). In brief, the experiment included 2 rainfall treatments (ambient and drought) × 4 spider treatments (Control, SW spider, AH spiders and SW + AH spider treatment) × 10 replicates, producing a total of 80 microcosms.

### Sample collection and analysis

To determine initial densities of soil fauna in the microcosms, we collected a 0.01-m^2^ (10 × 10 cm) litter sample from each microcosm before the spiders were added. One litter sample was taken from a random location from each microcosm at the beginning of the experiment in March 2012. Macro-invertebrates were first collected by hand, then the remaining soil fauna were extracted from the litter samples using Tullgren (“Berlese”) funnels for 7 d^49^ and collected into 90% ethanol. Fauna were identified to taxonomic groups according to Yi^50^ and counted under a microscope.

At two-month intervals over the course of a year, one litter bag was retrieved from each microcosm, sealed into a polyethylene bag and immediately returned to the laboratory. There, all soil fauna from the litter bags were extracted in Tullgren funnels and preserved in jars with 90% ethanol. Litter was then rinsed with distilled water to remove adhering soil. Then litter materials were dried in an oven (60 °C) to constant weights to determine remaining dry mass.

Invertebrates from the samples were divided into macrofauna and mesofauna according to Swift *et al.*^48^. We classified the macrofauna to taxonomic Orders, with the main Orders found being Psocoptera, Diplopoda, Diptera (larvae), Coleoptera (adults and larvae), Thysanoptera, Isopoda, Dermaptera, Isoptera, Blattodea and Symphyla. For analyses comparing densities of different taxonomic groups, we used the two most abundant groups (Psocoptera and Coleoptera) and aggregated the remaining groups as Other Macrofauna. The mesofauna from each sample were mainly Collembola and we identified and sorted Collembola to the genus level: primarily *Entomobrya* and *Paronellidae*. We aggregated *Sminthurinus*, *Onychiurus*, *Isotoma*, and *Neanura* into Other Collembola. We identified and sorted Acari to Oribatida and Other Acari, but Acari and ant are not potential prey for the spiders we studied^21^.

Soil moisture in microcosms was measured monthly at 0 to 5 cm depths using a FieldScout^®^ TDR 300 (Spectrum Technologies Inc., USA).

### Statistical analysis

First, we performed one-way ANOVA to detect differences in initial densities of soil fauna among treatments. To account for multiple comparisons, we used Tukey’s HSD to test for differences among spider treatments. Then we used a repeated measures Generalized Linear Mixed Model analysis with a Poisson error and a log-link function to assess differences among taxonomic groups (Macrofauna and Mesofauna) response to these experimental treatments. The independent variables included in the analysis were treatment and sampling period. In this analysis, microcosm was treated as a random effect with temporal autocorrelation (first-order autoregressive process) between samples. The Generalized Linear Mixed Models were performed with SPSS statistical software ver. 20.0 using the GENLIN procedure. Quasi-likelihood models were used to deal with overdispersion.

Leaf mass loss rate (k) from the litter bags was estimated using Olson’s formula^51^: X_t_ = X_0_ · e^−kt^, where X_t_ is mass remaining at time t, X_0_ was mass at t = 0, and k is annual mass loss rate. Then we estimated the strength and sign of trophic cascade as: trophic cascade index = (k_spider_ − k_control_)/k_control_, where k_spider_ means k value in either SW, AH or SW + AH treatment and k_control_ means k value in the control treatment^16^. We then used one-sample t-tests to examine difference between the trophic cascade indexes of three treatments, including SW, AH, SW + AH, and no-spiders control^16^. We present Bonferroni-corrected *P*-values.

## Additional Information

**How to cite this article**: Liu, S. *et al.* Spider foraging strategy effects on trophic cascades under natural and drought conditions. *Sci. Rep.* **5**, 12396; doi: 10.1038/srep12396 (2015).

## Supplementary Material

Supplementary Information

## Figures and Tables

**Table 1:** The abundance of Acari, not being potential prey of these spiders, from litter bags among four treatments under drought and ambient conditions (Mean ± s.e.m, n = 10).

Rainfall	Treatment	Oribatida	Other Acari
Drought	SW + AH	9.05 ± 0.43cd	6.64 ± 0.36b
	SW	11.67 ± 0.48b	6.61 ± 0.36b
	AH	11.01 ± 0.46bc	7.57 ± 0.38b
	Control	8.45 ± 0.40d	7.18 ± 0.37b
Ambient	SW + AH	12.10 ± 0.50b	7.27 ± 0.38b
	SW	13.27 ± 0.52b	7.29 ± 0.39b
	AH	15.86 ± 0.59a	9.47 ± 0.43a
	Control	13.07 ± 0.53b	9.59 ± 0.43a

Different letters in a column indicate significance among different treatments, at *P* < 0.05.
