# Supplementary material for: Spider foraging strategy affects trophic cascades under natural and drought conditions
Source: Sci Rep. 2015 Jul 23;5:12396. doi: 10.1038/srep12396 (PMC4511865; doi:10.1038/srep12396)
Supplement: Supplementary Information [file srep12396-s1.pdf]

Spider foraging strategy affects trophic cascades under natural and drought conditions

Shengjie Liu[1, 2], Jin Chen[1], Wenjin Gan[1], Douglas A. Schaefer[1], Jianmin Gan[1], Xiaodong Yang[1*]

[1]Key Laboratory of Tropical Forest Ecology, Xishuangbanna Tropical Botanical Garden, Chinese Academy of Sciences, Mengla, Yunnan 666303, China

[2]Key Laboratory of Vegetation Restoration and Management of Degraded Ecosystems, South China Botanical Garden, Chinese Academy of Sciences, Guangzhou 510650, China

[*]Corresponding author. Xiaodong Yang.

Postal address: Key Laboratory of Tropical Forest Ecology, Xishuangbanna Tropical Botanical Garden, Chinese Academy of Sciences Menglun, Mengla, Yunnan 666303, China.

Telephone: +86-691 8713236;

Fax: +86-691 8715070;

E-mail: yangxd@xtbg.ac.cn

Table S1. Initial densities of soil fauna populations among four treatments (mean ± s.e.m, n = 10)

Rainfall	Treatment	*Entomobrya*	*Paronellidae*	Other Collembola	Collembola	Psocoptera	Coleoptera	Other Macrofauna	Macrofauna	Oribatida	Other Acari
Drought	SW + AH	0.90 ± 0.41	3.40 ± 1.14	11.20 ± 1.72	15.40 ± 2.82	0.90 ± 0.27	1.40 ± 0.40	5.40 ± 1.79	7.40 ± 1.69	23.21 ± 7.57	27.38 ± 9.94
	SW	1.20 ± 0.59	3.90 ± 1.23	12.50 ± 2.59	14.60 ± 3.02	0.85 ± 0.31	1.80 ± 0.47	4.20 ± 1.80	9.50 ± 3.12	25.17 ± 8.17	19.57 ± 6.72
	AH	0.70 ± 0.13	3.50 ± 1.45	16.60 ± 3.12	21.80 ± 3.29	0.73 ± 0.15	1.70 ± 0.30	5.30 ± 1.02	8.30 ± 0.87	23.35 ± 7.60	25.50 ± 8.26
	Control	0.83 ± 0.13	3.13 ± 0.55	13.25 ± 1.87	18.25 ± 1.75	0.88 ± 0.52	2.25 ± 0.62	6.63 ± 1.31	9.75 ± 2.06	18.44 ± 6.79	30.03 ± 11.00
Ambient	SW + AH	0.70 ± 0.30	4.10 ± 0.95	11.20 ± 3.74	15.80 ± 4.43	1.00 ± 0.42	1.50 ± 0.54	4.90 ± 0.97	7.20 ± 1.41	24.60 ± 8.06	22.23 ± 7.68
	SW	0.80 ± 0.15	4.00 ± 1.01	10.50 ± 2.02	14.60 ± 2.43	1.10 ± 0.41	2.10 ± 0.74	4.80 ± 0.89	8.00 ± 1.20	22.53 ± 7.80	31.24 ± 10.08
	AH	0.92 ± 0.15	3.44 ± 0.58	12.44 ± 2.43	16.11 ± 2.42	0.89 ± 0.42	1.56 ± 0.41	4.56 ± 1.28	7.00 ± 1.88	22.26 ± 7.67	23.49 ± 8.64
	Control	0.95 ± 0.27	3.30 ± 0.83	12.00 ± 1.37	17.60 ± 1.81	0.90 ± 0.31	1.80 ± 0.79	5.30 ± 3.26	10.50 ± 4.15	25.11 ± 8.16	21.11 ± 7.23

Initial soil fauna did not significantly differ among treatments ($P > 0.05$)

Table S2. Summary of repeated measures GLMs used to test the effects of spider treatment, rainfall (drought and ambient treatments) and sampling period, and their interaction terms on the abundance of different soil fauna taxa (n = 10).

Source	*Entomobrya*		*Paronellidae*		Other Collembola		Total Collembola		Psocoptera		Coleopera		Other Macrofauna		Total Macrofauna	
	Wald x^2	*df*	Wald x^2	*df*	Wald x^2	*df*	Wald x^2	*df*	Wald x^2	*df*	Wald x^2	*df*	Wald x^2	*df*	Wald x^2	*df*
Sampling period (S)	311.46***	5	1091.62***	5	934.54***	5	3300.64***	5	29.75**	5	48.89***	5	41.65**	5	218.40***	5
Treatment (T)	18.01**	3	6.34	3	15.98**	3	24.28***	3	8.63*	3	8.77*	3	17.80*	3	2.17	3
Rainfall (R)	13.57**	1	10.32***	1	156.33***	1	165.68***	1	135.08***	1	21.11**	1	58.09**	1	7.85*	1
T × S	57.28***	27	223.63***	27	38.30***	27	242.90***	27	43.35***	27	20.99**	27	13.75*	27	48.13**	27
T × R	18.30**	3	40.17**	3	11.11*	3	19.29***	3	6.23*	3	16.43**	3	29.09**	3	18.54*	3
T × R × S	9.09	20	6.60	20	6.95	20	1.14	20	4.51	20	0.93	20	1.47	20	2.06	20

(*P = 0.05, **P = 0.01, and ***P = 0.001)

Table S3. Leaf-litter fall in this tropical secondary forest and amount added to microcosms per month

	May	Jun.	Jul.	Aug.	Sep.	Oct.	Nov.	Dec.	Jan.	Feb.
Litter fall in natural forest (g/m^2)	114.99	122.14	97.73	160.45	110.84	253.99	68.54	20.25	94.52	45.54
Litter fall addition in mesocosm (g)	32.50	34.52	27.62	45.34	31.32	71.78	19.37	5.72	26.71	12.87

Table S4. The number of spider replenished in different treatment microcosm during the experiment (mean ± s.e.m, n = 10).

Rainfall	Treatment	Sit-and-wait spiders	Actively hunting spiders
Drought	SW + AH	0.18 ± 0.04	0.22 ± 0.05
	SW	0.27 ± 0.06	-
	AH	-	0.32 ± 0.04
Ambient	SW + AH	0.22 ± 0.04	0.26 ± 0.04
	SW	0.28 ± 0.05	-
	AH	-	0.33 ± 0.05

Fig S1. Schematic of the detrital food web with the relevant groups, their functions and the relationships among them, especially the microbi-detritivores.

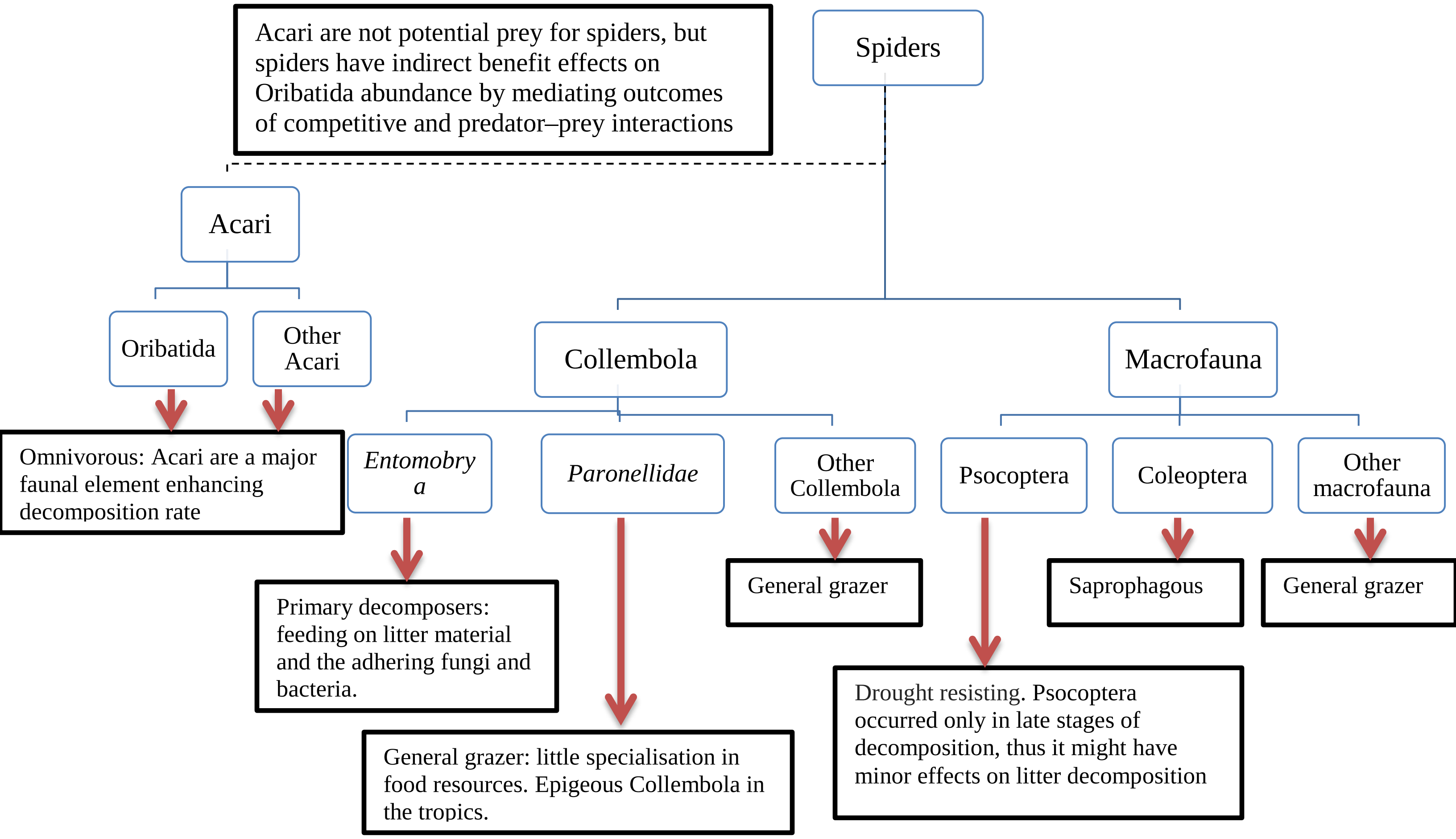

Fig. S2. Annual (A) and inter-annual (B) variations of rainfall from 2002 to 2012 in Xishuangbanna, southwest of China. Dotted line indicates long-term average annual precipitation (1557 mm).

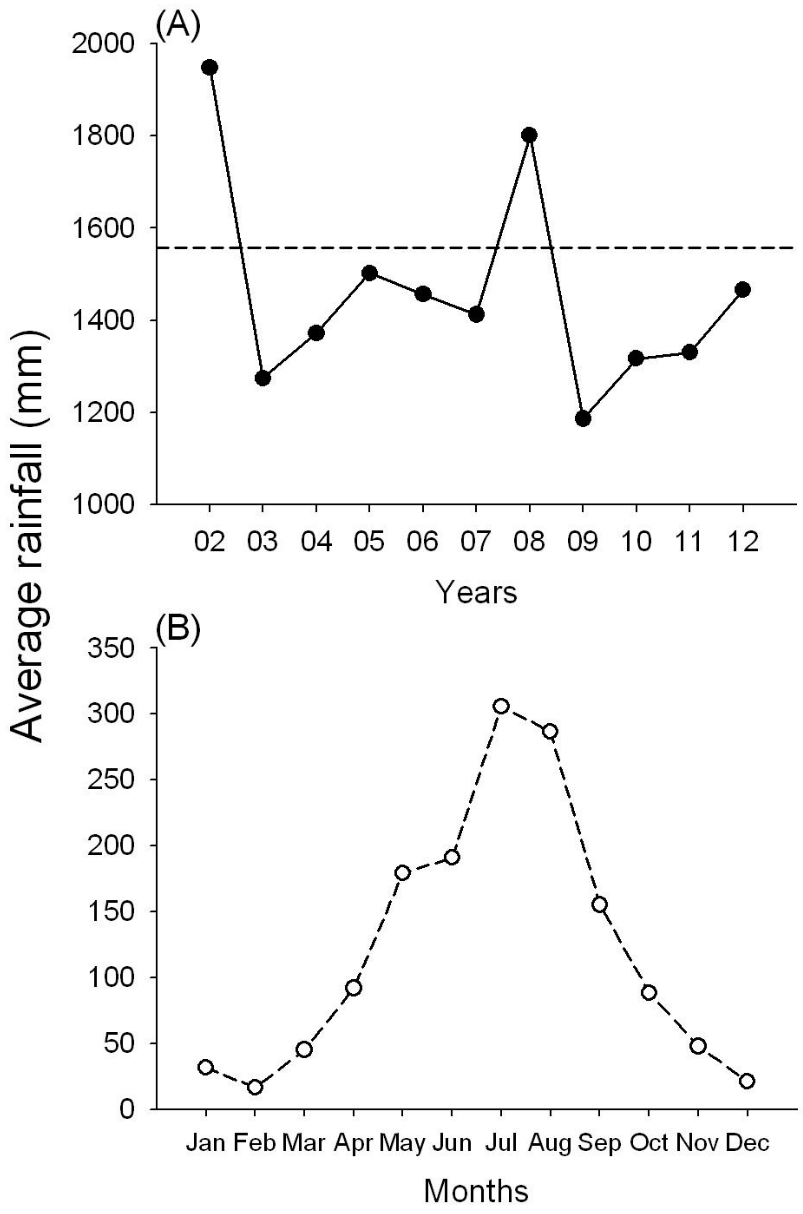

Fig. S3. Monthly measures of volumetric water content in microcosms under drought and ambient conditions (mean ± s.e.m, n = 40)

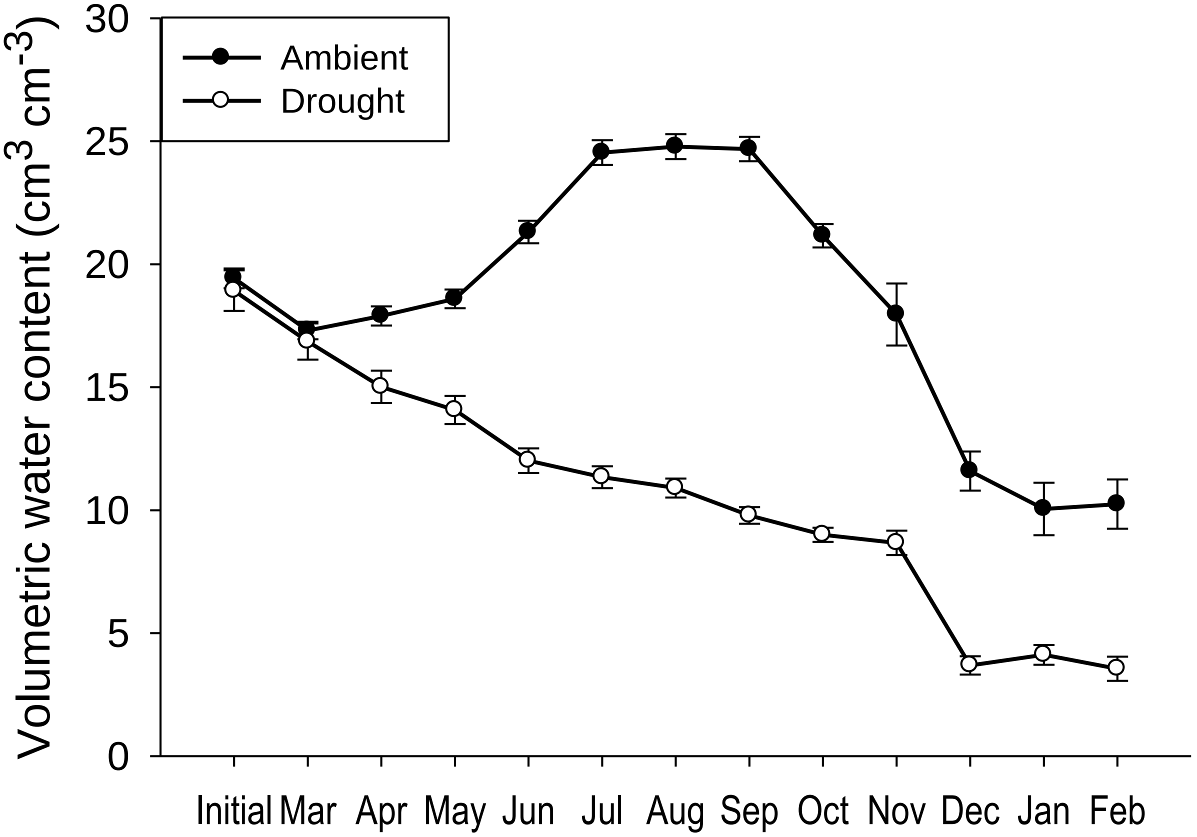

Fig. S4. Changes in litter mass remaining in different spider treatments over time in ambient and drought conditions. (mean ± s.e.m, n=10)

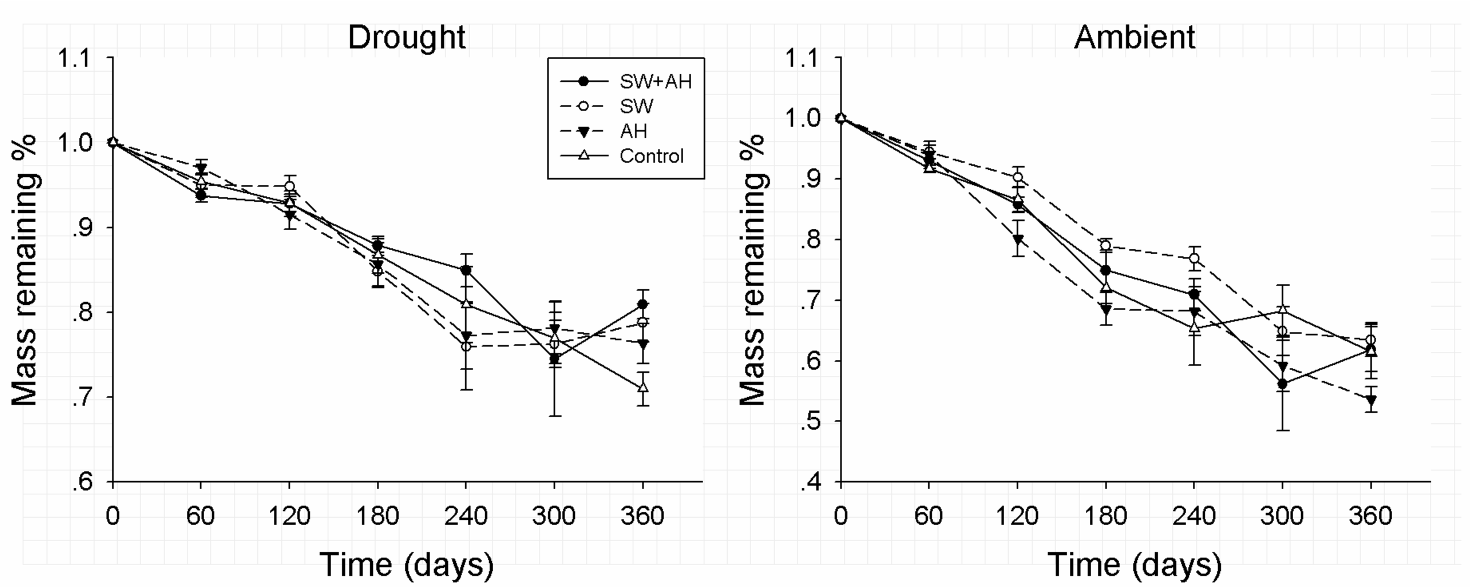

Fig description: Under ambient rainfall conditions, actively hunting spider treatment had lower litter mass remaining (higher mass loss) compared with control treatment, especially at 120, 180 and 360 days. But mass remaining in sit-and-wait spider treatment was not different from the control treatment.

Under drought conditions, there were significantly lower masses remaining in control treatment than that in the other three spider treatments at last sampling (360 days).

Fig. S5. Predation on Collembola by different foraging strategies spider in microcosms (mean ± s.e.m, n=10).

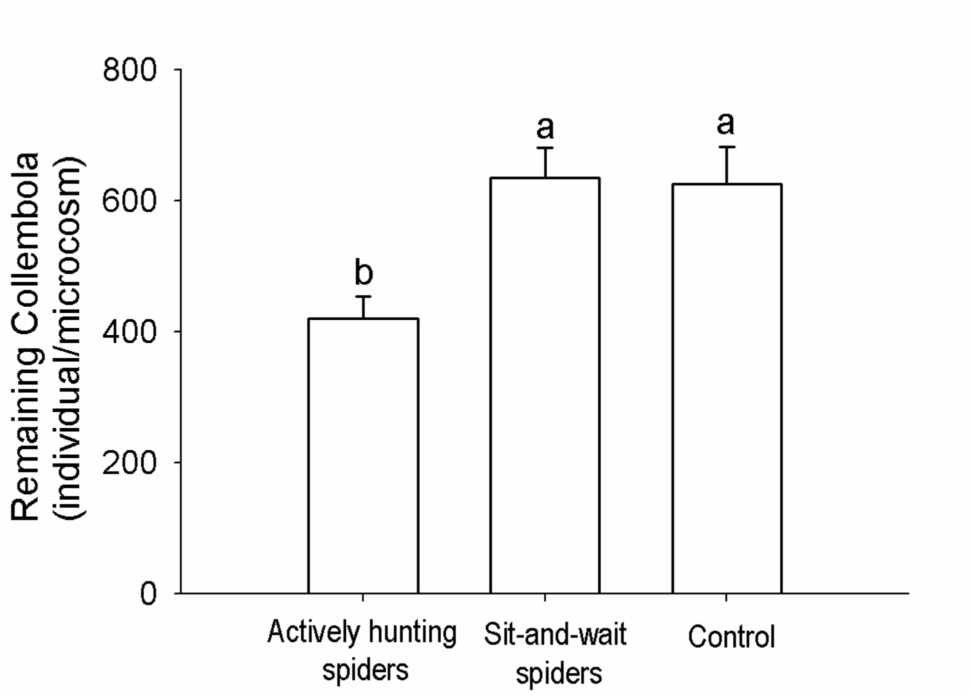

Fig description: Sit-and-wait spiders are adult female *Macrothele yunnanica*; actively hunting spiders are adult female *Pardosa laura*. The prey of spider are Collembola species (*Seira delamarei Jacquemart*) in each microcosm. Different foraging strategies had different predator effects on Collembola, with control lacking all spiders.
